# Supplementary material for: Significant increase in eco-efficiency of China’s grain production from 2000 to 2022: Trend changes, typological evolution, and driving factors
Source: PLoS One. 2025 Sep 19;20(9):e0332740. doi: 10.1371/journal.pone.0332740 (PMC12448343; doi:10.1371/journal.pone.0332740)
Supplement: Attachment — Submitted filename: Response to Reviewers.pdf [file pone.0332740.s002.pdf]

Response to Reviewers

Dear Editor,

Thank you for your attention and guidance regarding our manuscript. We have carefully considered the reviewers' comments and found them to be extremely helpful. Based on the specific suggestions from the editorial office and the reviewers, we have thoroughly revised the manuscript and included a point-by-point response to each comment below for your review. For your convenience, the manuscript number is PONE-D-24-43272.

Thank you once again to the editor and reviewers for their review and support.

Author

August 28, 2025

Academic Editor

1. Please ensure that your manuscript meets PLOS ONE's style requirements, including those for file naming. The PLOS ONE style templates can be found at https://journals.plos.org/plosone/s/file?id=wjVg/PLOSOne_formatting_sample_main_body.pdf and https://journals.plos.org/plosone/s/file?id=ba62/PLOSOne_formatting_sample_title_authors_affiliations.pdf

【Revision Notes】 Thank you very much for providing the PLOS ONE template link and pointing out the formatting and file-naming requirements. We have thoroughly checked and revised the manuscript based on the two official templates, and have fully reflected the corresponding adjustments in both the revised manuscript and the marked-up revision. At the same time, we have standardized and normalized all file names according to PLOS ONE requirements, using informative English file names and avoiding spaces and special characters. If the editorial office has any further specifications or preferences regarding layout or naming, we will promptly make the necessary revisions. Thank you again for your clear guidance and support; we believe these improvements have significantly enhanced the manuscript’s compliance and readability.

2. PLOS requires an ORCID ID for the corresponding author in Editorial Manager on papers submitted after December 6th, 2016. Please ensure that you have an ORCID iD and that it is validated in Editorial Manager. To do this, go to ‘Update my Information’ (in the upper left-hand corner of the main menu), and click on the Fetch/Validate link next to the ORCID field. This will take you to the ORCID site and allow you to create a new iD or authenticate a pre-existing iD in Editorial Manager.

【Revision Notes】 Thank you very much for the reminder and the detailed instructions regarding the ORCID verification requirement. We have carefully read the guidance you provided and followed it strictly: the corresponding author has confirmed possession of a valid ORCID iD and has, via the Editorial Manager main menu’s “Update my Information” entry in the upper left, clicked the “Fetch/Validate” link next to the ORCID field to be redirected to the ORCID website to complete authorization and verification. The system has successfully linked that ORCID iD to the Editorial Manager account. We thank the reviewer again for the thorough guidance and hereby confirm that all related actions have been completed.

3. Thank you for stating the following financial disclosure: “This work was funded by the National Social Science Foundation of China (NSSFC) Project “Research on the Coordinated Promotion of Agricultural Emission Reduction, Carbon Sequestration, and Food Security under the Background of ‘Double Carbon’ Goals” (No. 22AJY008).” Please state what role the funders took in the study. If the funders had no role,

please state: “The funders had no role in study design, data collection and analysis, decision to publish, or preparation of the manuscript.” If this statement is not correct you must amend it as needed. Please include this amended Role of Funder statement in your cover letter; we will change the online submission form on your behalf.
**【Revision Notes】** Thank you for your reminder and guidance regarding the funding statement. This work was funded by the National Social Science Foundation of China (NSSFC) Project “Research on the Coordinated Promotion of Agricultural Emission Reduction, Carbon Sequestration, and Food Security under the Background of ‘Double Carbon’ Goals” (No. 22AJY008). The funders had no role in study design, data collection and analysis, decision to publish, or preparation of the manuscript. We again thank the reviewers for their careful review and professional suggestions.
4. When completing the data availability statement of the submission form, you indicated that you will make your data available on acceptance. We strongly recommend all authors decide on a data sharing plan before acceptance, as the process can be lengthy and hold up publication timelines. Please note that, though access restrictions are acceptable now, your entire data will need to be made freely accessible if your manuscript is accepted for publication. This policy applies to all data except where public deposition would breach compliance with the protocol approved by your research ethics board. If you are unable to adhere to our open data policy, please kindly revise your statement to explain your reasoning and we will seek the editor's input on an exemption. Please be assured that, once you have provided your new statement, the assessment of your exemption will not hold up the peer review process.
**【Revision Notes】** Thank you very much for reminding us about the timing of data availability and sharing. We fully support and comply with PLOS ONE's open data policy and confirm that all data from this study will be made publicly available without access restrictions immediately after the manuscript is accepted. To avoid delaying publication, our data-sharing plan has been clarified and is being implemented in parallel: metadata and reproducibility materials have already been prepared so that the data and related materials can be posted to the designated public repository upon acceptance.
5. We note that Figure 2 in your submission contain map images which may be copyrighted. All PLOS content is published under the Creative Commons Attribution License (CC BY 4.0), which means that the manuscript, images, and Supporting Information files will be freely available online, and any third party is permitted to access, download, copy, distribute, and use these materials in any way, even commercially, with

proper attribution. For these reasons, we cannot publish previously copyrighted maps or satellite images created using proprietary data, such as Google software (Google Maps, Street View, and Earth). For more information, see our copyright guidelines: http://journals.plos.org/plosone/s/licenses-and-copyright.<br>We require you to either (1) present written permission from the copyright holder to publish these figures specifically under the CC BY 4.0 license, or (2) remove the figures from your submission:
**【Revision Notes】** Thank you very much for your reminder and detailed explanation regarding map imagery copyright. We have fully understood and respect PLOS ONE's copyright and licensing policies. To fully comply with the journal' s requirements and avoid any potential copyright risks, we have taken the following measure (2): Figure 2 and its corresponding caption and citation have been completely removed from the manuscript and related submission materials, and the text and numbering in the manuscript have been adjusted accordingly (the original Figure 3 and subsequent figures/tables have been renumbered and moved forward to maintain continuity).
6. Please remove all personal information, ensure that the data shared are in accordance with participant consent, and re-upload a fully anonymized data set. Note: spreadsheet columns with pers onal information must be removed and not hidden as all hidden columns will appear in the publish ed file. Additional guidance on preparing raw data for publication can be found in our Data Policy (https://journals.plos.org/plosone/s/data-availability#loc-human-research-participant-data-and-other-sensitive-data) and in the following article: http://www.bmj.com/content/340/bmj.c181.long.
**【Revision Notes】** Thank you for your strict requirements and clear guidance regarding data privacy and anonymization. We have completed a comprehensive review of the submitted materials and implemented the following corrections: all information that could identify individuals has been removed; the scope of data sharing was checked item-by-item against participants' informed consent forms to ensure publicly released data strictly complies with consent provisions; the raw data underwent a further anonymization check, confirming there are no direct identifiers or indirect identifiers that could be re-identified through linkage with other information. To eliminate any residual risk, we have "deleted (not merely hidden)" all spreadsheet columns containing personal information or that could lead to re-identification, ensuring that no hidden columns or metadata leaks will appear in the released version. We again thank the editor and reviewers for their professional advice and patient guidance. We will continue to follow the journal's data-sharing and privacy-protection policies to ensure that data are open, secure, and reproducible.

7. Please include captions for your Supporting Information files at the end of your manuscript, and update any in-text citations to match accordingly. Please see our Supporting Information guidelines for more information: http://journals.plos.org/plosone/s/supporting-information.

【Revision Notes】 Thank you very much for your review and guidance. After verification and a full read-through, we confirm that no Supporting Information needs to be provided for this manuscript. The reasons are as follows: First, the data used in this study, variable definitions, model specifications, and estimation results are all fully presented in the main text, tables, and figures; second, the study does not involve questionnaires, interview records, code scripts, or external raw datasets that would need to be attached separately; third, to ensure reproducibility, key parameter settings, data sources, and processing procedures are described in detail in the "Methods and Data" and "Results and Discussion" sections, with no additional supplementary materials to provide. Based on the above, we have not included a Supporting Information section at the end of the manuscript, nor are there any in-text references that require updating. If the journal requires a formal "no SI" statement, we can add a brief note at the end of the manuscript: "No Supporting Information is provided for this manuscript." If you still recommend providing any specific type of supplementary material, we will promptly prepare it and submit it in PLOS ONE format.

8. If the reviewer comments include a recommendation to cite specific previously published works, please review and evaluate these publications to determine whether they are relevant and should be cited. There is no requirement to cite these works unless the editor has indicated otherwise.

【Revision Notes】 Thank you for your reminder and guidance. We base decisions to cite literature on the relevance to our research and on academic rigor, not on formalistic or compulsory requirements. In this round of review, the reviewers provided three specific citation details (including author, title, or DOI); accordingly, we have added citations to these three works in the revised manuscript. At the same time, we have again conducted a comprehensive review of the existing references to ensure sufficient and appropriate coverage of key areas and the latest developments.

9. Please review your reference list to ensure that it is complete and correct. If you have cited papers that have been retracted, please include the rationale for doing so in the manuscript text, or remove these references and replace them with relevant current references. Any changes to the reference list should be mentioned in the rebuttal letter that accompanies your revised manuscript. If you need to cite a retracted article, indicate the article's retracted status in the References list and also include a citation and full reference for the retraction

notice.
**【Revision Notes】** Thank you for your reminder regarding the completeness and accuracy of the references. We have conducted a comprehensive check and update of the references accordingly. First, we verified each citation's authors, title, journal information, volume/issue/pages, year, and DOI, and corrected individual inconsistencies in formatting and punctuation to ensure uniform, properly searchable citations. Second, we cross-checked the references against journal websites and major literature databases (including retraction databases and notices) and confirmed that no retracted papers were found among the manuscript's references; therefore, there is no need to explain citations of retracted literature in the main text or to replace any references. At the same time, in response to this round of review comments we added three relevant references and inserted citations at the corresponding positions in the main text; the reference list and citation numbering have been updated accordingly to ensure one-to-one correspondence with the text. All of the above changes have been faithfully recorded in the revised manuscript. We again thank the editor and reviewers for their guidance in helping us ensure the references' completeness, accuracy, and compliance.
Additional Editor Comments: Both reviewers found the manuscript to be technically sound and well-structured, recommending minor revisions. I concur with their assessment and require that the authors address the specific points raised, particularly refining the conceptual explanation of eco-efficiency, incorporating the suggested citations, clarifying methodological descriptions, and expanding the discussion on policy implications and data limitations. These adjustments are necessary for acceptance. Minor typographical corrections and improvements to figure captions are recommended for clarity.
**【Revision Notes】** We appreciate the editor's summary and affirmation of the two reviewers' comments. We thank you for the overall assessment that the manuscript is technically reliable and structurally sound. In accordance with the suggestion to "make minor revisions," we have carried out comprehensive and targeted revisions. In response to the key points emphasized by the editor (clarifying the concept of ecological efficiency, incorporating the recommended references, elaborating methodological descriptions, expanding the discussion of policy implications and data limitations, and making minor typographical corrections and improvements to figure captions), we have made the corresponding adjustments and indicated changes in the revised manuscript. We will continue to cooperate with the journal's copyediting process to ensure that the manuscript's quality and presentation meet publication standards. Thank you again to the editor and reviewers for their careful guidance and constructive suggestions.

Reviewer 1

1. Eco-Efficiency Evolution Concept: The manuscript discusses the evolution of eco-efficiency but could benefit from a more detailed re-explanation of this concept. A clearer definition and contextualization within the broader sustainability framework would strengthen the discussion.

【Revision Notes】 We sincerely thank you for your valuable suggestions. In the revised manuscript, we have explicitly stated that the concept of eco-efficiency originates from the theory of sustainable development and was proposed by the World Business Council for Sustainable Development in the 1990s. We have also elaborated on its core essence as a bridge connecting economic growth and environmental protection. Furthermore, we have systematically reviewed the evolution of eco-efficiency from theory to application, demonstrating its development process from qualitative description to quantitative evaluation, as well as its application evolution process from the industrial sector to multiple industries. In particular, in the field of food production, we have emphasized the shift in research perspective from purely yield-oriented to resource and environmental efficiency-oriented, and the theoretical deepening process from considering a single environmental factor to comprehensively considering multi-dimensional ecological and environmental factors. These revisions have strengthened the theoretical foundation of the paper, providing a more solid theoretical support for the subsequent empirical analysis. Thank you again for your suggestions, which have given us the opportunity to further improve the quality of the paper.

【Specific modifications are as follows】

The concept of ecological efficiency emerged from sustainable development theory and was formally introduced by the World Business Council for Sustainable Development (WBCSD) in the 1990s, with its core principle defined as “creating more economic value with less resource consumption and environmental impact.” Serving as a critical bridge between economic growth and environmental protection, ecological efficiency has become a key instrument for achieving the United Nations Sustainable Development Goals (UN SDGs). As global sustainability challenges become increasingly severe, both theoretical research and practical applications of this concept have demonstrated a rapidly growing trend. Scholars have delved into the internal characteristics of eco-efficiency [11], the goal requirements for eco-efficiency [12], the realization paths for eco-efficiency [13], and other detailed theoretical discussions. Moreover, they have developed index systems to quantitatively assess eco-efficiency based on its theoretical implications [14,15]. As research progresses, scholars have begun to examine

eco-efficiency issues in various fields, including arable land [16], farming [17], livestock [18], the marine industry [19], agriculture [20], industry [21], and services [22]. Among these, eco-friendly advancement of cereal cultivation, a crucial area related to national livelihood, has garnered significant attention.

Early studies on grain production primarily concentrated on supply quantity and price stabilization [23]. With the emergence of sustainable development principles, scholars have progressively recognized that development models solely pursuing yield increases while neglecting resource and environmental costs are no longer viable. The research focus in food production has shifted from traditional yield-oriented approaches to resource and environmental efficiency orientation, with the ecological efficiency of food production increasingly gaining scholarly attention [8].

Within the sustainable development framework, research on ecological efficiency in food production has evolved from a single-dimensional approach to multi-dimensional integration. In recent years, scholars have conducted a series of studies examining carbon emission efficiency in food production [24], environmental efficiency of food production [25], and green total factor productivity in food systems [26], while exploring their patio-temporal heterogeneity. This evolution reflects the theoretical deepening process of sustainable food production research from considering single environmental factors to comprehensive assessment of multiple ecological and environmental dimensions. These theoretical explorations and empirical studies have significantly enriched the research content on sustainable food production development and laid the foundation for constructing a more comprehensive ecological efficiency evaluation system for food production.

Among the various indicators used to assess the sustainable development of grain production, eco-efficiency has garnered significant attention for its ability to comprehensively evaluate both economic output and environmental impact. Now, the primary methods for assessing the eco-efficiency of grain production include LCA (Life Cycle Assessment), SFA (Stochastic Frontier Analysis) and DEA (Data Envelopment Analysis). LCA is an effective method for measuring the environmental impact of the entire grain production process, with a particular emphasis on carbon emissions [27]. Data Envelopment Analysis (DEA) models are widely employed due to their ability to handle multiple inputs and outputs without the need for a specific production function form [28]. However, researchers employing DEA models to assess grain production diverge in their selection of undesirable output indicators. While the majority of scholars consider surface source pollution in grain production to be an undesirable output [29], only a limited number of studies have included carbon emissions as an undesired output

[26]. In light of the pressing need to reduce greenhouse gas emissions in agriculture, it is crucial to consider both surface source pollution and carbon emissions in the grain production process when evaluating eco-efficiency.

2. Citations for Supporting Evidence: Line 24: Consider citing [https://doi.org/10.1016/j.plaphy.2025.109574] to support discussions on the physiological mechanisms driving eco-efficiency improvements. Lines 35-39: Additional citations, such as [https://doi.org/10.3389/fpls.2022.863760] and [https://doi.org/10.3389/fnut.2024.1348235], should be incorporated to reinforce key arguments on agronomic practices and nutritional impacts on eco-efficiency.

【Revision Notes】 Thank you for suggesting the inclusion of additional literature. As requested, we have added citations to these three important references in the target paragraph, with particular emphasis on the significant role of agronomic optimization (Asdullah et al., 2024) and innovative nutrient management (Ahmed et al., 2022) in enhancing eco-efficiency. In the revision, we highlighted that these studies offer important insights for improving eco-efficiency in agricultural production, while also defining our study's focus on the dynamic evolution and driving mechanisms of eco-efficiency from a macro perspective. This revision not only enriches the literature background but also maintains the coherence of the research's main line, better highlighting the theoretical contributions and practical significance of this study in the field of grain production eco-efficiency.

3. Typological Evolution Analysis: The paper effectively categorizes typological changes, but providing more comparative examples or case studies could further illustrate key shifts in production systems.

【Revision Notes】 Thank you for your valuable suggestions on this paper. Based on your feedback, we have added case studies of three representative provinces in Section 3.4, representing major grain-producing areas (Heilongjiang Province), major grain-consuming areas (Guangdong Province), and areas with balanced production and consumption (Guangxi Province). These cases provide a detailed analysis of the key characteristics, driving mechanisms, and major challenges faced by the transformation of grain production systems in different functional regions.

These additional comparative cases make the analysis of production system transformation more concrete and differentiated, helping readers to deeply understand the intrinsic mechanisms of the evolution of grain production eco-efficiency in different functional regions. Through the comparative analysis of these three typical cases, we reveal the structural differences in resource endowments, institutional environments, and market conditions among different functional regions, and how these differences affect their

transformation paths and outcomes. At the same time, we also distilled transferable experiences and models such as policy-technology-scale synergistic driving, and the integration of appropriate scale with circular agriculture, providing more targeted empirical evidence for building a differentiated regional policy system. This modification not only responds to your suggestion to add comparative cases, but also further enriches the empirical content of the article, enhancing its theoretical persuasiveness and policy relevance. We believe that these modifications will effectively improve the academic quality and application value of the article.

【Specific modifications are as follows】

3.4 Comparative Case Studies of Evolutionary Patterns in Production Systems Across Typical Regions

3.4.1 Case Study of Major Production Area: Analysis of Transformation Characteristics in Heilongjiang Province

As a strategic core area for national food security, Heilongjiang Province demonstrates a transformation pathway characterized by significant policy-technology-scale synergistic driving forces: First, leveraging key projects such as the Sanjiang Plain Agricultural Comprehensive Development, the province has established a trinity foundational support system integrating farmland protection, soil fertility enhancement, and water conservancy safeguards. Second, through its agricultural reclamation system, the province has constructed a comprehensive "R&D-extension-application" technology diffusion mechanism, substantially increasing the contribution rate of agricultural scientific and technological advancements. Third, by developing new-type agribusiness entities with scaled operations, the province has achieved enhanced efficiency in resource-intensive utilization, maintained relatively low fertilizer and pesticide application intensity per unit area, and demonstrated a synergistic development pathway that balances grain production increases with ecological protection.

3.4.2 Case Study of Major Consumption Area: Analysis of Development Constraints in Guangdong Province

With Guangdong Province's grain self-sufficiency rate declining to 25.4%, its ecological efficiency reflects the transformation dilemma facing highly urbanized regions: On one hand, the compression effect from industrialization and urbanization has intensified the industrialization-urbanization paradox, exacerbating both

diseconomies of scale and negative externalities in grain production, while small-scale farmers face constraints such as high adoption barriers for new technologies and insufficient incentives for green production practices. Examining the root causes reveals that: first, reforms in farmland property rights have lagged behind, and market allocation mechanisms for urban-rural construction land remain imperfect, resulting in the continuous loss of premium cropland; second, agricultural support policy instruments remain overly singular, failing to effectively address the structural contradictions characterized by small-scale, high-cost, and low-yield production.

3.4.3 Case Study of Production-Consumption Balanced Area: Exploring Transformation Models in Guangxi Province

The innovative value of Guangxi Province lies in exploring integration pathways between moderate-scale operations with circular agriculture: First, the province has constructed a “sugarcane-livestock farming-compost recycling” material circulation system, significantly improving nutrient cycling efficiency within agricultural production systems. Second, the province has developed county-level characteristic agricultural industrial clusters (such as selenium-rich rice industrial belts), enhancing value-added per unit of resource through industrial chain extension. Third, the province has implemented ecological agricultural models in karst landform areas, promoting three-dimensional agroforestry-pastoral systems combining “fruit trees-forage grass-livestock” in rocky desertification control zones, achieving synergistic improvement in both resource carrying capacity and production efficiency in ecologically vulnerable regions.

4. Policy and Practical Implications: While the study highlights driving factors, expanding on policy recommendations and their potential impact on future agricultural strategies in China would enhance the practical relevance of the findings.

【Revision Notes】 Thank you very much for your valuable feedback on this article. We have carefully studied your review comments and made targeted revisions and improvements to the conclusions and recommendations sections of the article. The main modifications are as follows:

First, we added a fifth point to the conclusion section, clearly articulating the potential impact of this study on China's future agricultural strategy. The revised content emphasizes the practical significance of the research results in the context of the "dual carbon" goals and the rural revitalization strategy, clarifying that improving the ecological efficiency of grain production is a key pathway to achieving the green and low-carbon transformation of agriculture.

Second, we highlighted the theoretical supporting role of the "club convergence" phenomenon and the "positive spillover" effect discovered in the research for building a regional coordinated development mechanism. We also strengthened the connection between this study and the national food security strategy and ecological civilization construction, thereby enhancing its practical application value.

Through the above revisions, the conclusions and recommendations sections of this article more prominently highlight the policy and practical significance. The recommendations put forward are also more specific and actionable, and the differentiated development strategies for different functional areas are more clearly defined, effectively addressing your valuable feedback. The revised content more fully reflects the application value and practical significance of this research, providing a more targeted policy basis for promoting the improvement of grain production ecological efficiency.

【Specific modifications are as follows】

6. Conclusions and recommendations

6.1 Conclusion

This research utilizes a combined approach of global super-efficiency SBM modeling and life cycle assessment (LCA) to analyze cereal cultivation environmental performance across 31 Chinese provinces from 2000 to 2022. By synthesizing conventional and spatial kernel density estimation techniques, the study investigates the dynamic patterns and long-term shifts in agricultural eco-efficiency distribution. The analysis identifies critical factors influencing environmental efficiency at various performance levels, considering holistic, temporal, and spatial dimensions. The findings indicate that:(1) The overall eco-efficiency level of grain production in China is relatively low, with a 31.8% potential for improvement and significant regional disparities following a spatial pattern of "major grain-producing regions > production-sales balance regions > the major grain-consuming regions" (2) While grain production eco-efficiency generally shows improvement, spatial polarization is evident. This is demonstrated by the gradual weakening of polarization in the major grain-producing regions, expansion in the major grain-consuming regions, and initial strengthening followed by weakening in the production-sales balance regions. (3) The eco-efficiency of grain production is characterized by "club convergence" and a "positive spillover" effect. (4) The primary drivers influencing grain production eco-efficiency evolve from natural endowment to scion-economic factors and then to technological progress. Across all levels of efficiency, gross total

agricultural output value, water resources endowment, and structure of agricultural production consistently exhibit a significant positive impact. This phenomenon underscores the cumulative advantage principle, wherein resource-rich entities progressively enhance their position, while resource-poor ones experience further decline. (5) This study, through the innovative integration of Life Cycle Assessment (LCA) and Global Super-efficiency SBM model, not only extends the research perspective on ecological efficiency evaluation of grain production, but also provides more scientifically robust methodological support for cross-temporal, multi-regional dynamic efficiency comparisons. The findings of this study hold significant implications for China's agricultural strategy; in the context of dual-carbon goals and rural revitalization, enhancing the ecological efficiency of grain production emerges as pivotal to agricultural green transformation. The “club convergence” and “positive spillovers” identified in this research provide theoretical foundations for constructing regional collaborative mechanisms.

6.2 Recommendations

The findings of the research study lead to the following recommendations:

First, Categorized guidance and region-specific implementation. (a) Major Production Areas: Leveraging green scale effects and consolidating ecological efficiency advantages. Establish agricultural technology integration demonstration bases in Heilongjiang, Jilin, and other regions; promote precision fertilization techniques; implement large-scale black soil conservation tillage; and establish crop rotation and fallow systems, allocating appropriate quantities of cropland for fallowing. (b) Major Consumption Areas: Developing high-quality, high-efficiency agriculture and accelerating ecological transformation. Construct several urban modern agricultural demonstration zones in Guangdong, Zhejiang, and other regions to improve output efficiency per unit area. (c) Production-Consumption Balanced Areas: Developing specialty ecological agriculture and constructing circular agricultural systems. Develop multiple specialty agricultural demonstration parks in Guangxi, Yunnan, and other regions to increase the growth rate of output value from specialty agricultural products.

Second, Regional coordination for holistic improvement. Establish a regional coordination mechanism led by the Ministry of Agriculture and Rural Affairs; convene regular joint conferences on collaborative development among the three functional area types; establish dedicated funds for regional ecological efficiency collaboration; and incorporate grain production ecological efficiency into government performance evaluations. Form a national expert committee on enhancing grain production ecological efficiency; implement the “Science and Technology

Commissioner Plus” initiative, deploying numerous technical personnel to serve on the front lines; establish an agricultural technology training system to conduct large-scale training programs for new-type professional farmers.

Third, policy coordination for systematic advancement. Establish a green and low-carbon agriculture subsidy system that incorporates reduction and efficiency enhancement into subsidy criteria; reform agricultural insurance policies by developing green crop insurance products; and establish an agricultural carbon sink trading mechanism. Continuously expand the area through high-efficiency water-saving irrigation; construct multiple regional grain logistics centers; and develop numerous rural clean energy demonstration villages. Establish a national green agriculture development fund; develop green agricultural credit products; and encourage the issuance of specialized green agricultural bonds. Implementation of these recommendations would likely ensure national food security while simultaneously reducing agricultural carbon emission intensity, improving utilization rates of fertilizers and pesticides, decreasing agricultural non-point source pollutant emissions, and providing robust support for green and low-carbon agricultural development.

Despite the meaningful findings generated by this study, certain limitations remain. First, the assumption of nationally uniform emission coefficients may underestimate the effects of regional heterogeneity. Second, long time-series data may be subject to inconsistencies in statistical methodologies and measurement errors. Finally, provincial-level analysis may mask efficiency disparities among different areas within provinces. Future research could enhance study quality by developing regionally differentiated emission coefficient systems and employing finer-grained data at municipal and county levels.

5. Minor Comments: Some minor typographical and grammatical corrections are needed for better readability. Consider improving the clarity of figure captions and ensuring that all abbreviations are defined upon first use.

【Revision Notes】Thank you very much for your review and constructive comments on our manuscript. Regarding the "minor suggestions," we have thoroughly reviewed and revised the manuscript, as detailed below: Firstly, concerning typesetting and grammar corrections, we have proofread and polished the entire text paragraph by paragraph, correcting minor grammar, punctuation, and tense inconsistencies. We have also standardized heading levels, paragraph indentation, table and figure caption formats, and reference citations. Longer sentences have been appropriately split and structurally optimized to improve overall readability and logical coherence. Secondly, regarding figure legend clarity, we have standardized symbols, line types, colors, and label meanings, ensuring consistency for the same variable across different figures. We have increased

the font size and contrast of figure legends and annotations, and added textual explanations to legend items where necessary. Simultaneously, we have optimized the layout and white space of figure-notes-legends to ensure clarity under both black-and-white printing and screen viewing conditions; individual figures have also been updated with high-resolution versions. Finally, regarding first-time definition and consistency of abbreviations, we have reviewed the entire text to ensure that abbreviations are defined in full with the complete term and abbreviation in parentheses upon their first appearance (e.g., Life Cycle Assessment (LCA)), and that the abbreviation is used consistently thereafter. Thank you again for the reviewer's meticulous suggestions.

Reviewer 2

This study assessed the eco-efficiency of grain production in 31 Chinese provinces from 2000 to 2022 using a global super-efficiency SBM model combined with life cycle assessment (LCA) methodology. The authors analyzed temporal and spatial dynamics, distributional evolution, and spatial spillover effects. Additionally, quantile regression is employed to identify region- and time-specific drivers of eco-efficiency at different performance levels. There are some important concerns to be addressed befor its worthy for the publication.

1. While the integration of the global super-efficiency SBM model and LCA is valuable, the authors should more clearly highlight how this methodological integration advances prior research beyond incremental improvement.

【Revision Notes】 Thank you very much for your valuable comments on this paper. We have revised the research methods section to elaborate on the three methodological advances achieved by combining life cycle assessment (LCA) with the global super-efficiency SBM model. The revisions are mainly concentrated in Section 2.2, where we emphasize the innovative value of this methodological integration, and explain in detail how it breaks through the prevailing incremental research paradigm in the literature along three dimensions: assessment scope (from a single environmental factor to full life-cycle), temporal span (from static cross-sections to dynamic evolution), and policy precision (from general recommendations to differentiated guidance).

【Specific modifications are as follows】

This research achieves a triple methodological breakthrough by integrating Life Cycle Assessment (LCA) with a Global Super-efficiency SBM model: Firstly, the introduction of LCA addresses the deficiency of traditional DEA models in quantifying undesirable outputs such as non-point source pollution and carbon emissions, establishing a "sowing-to-harvest" full-chain environmental footprint assessment system that enables comprehensive quantification of ecological impacts throughout the entire grain production process, rather than merely focusing on localized environmental effects of specific stages. Secondly, efficiency measurements with global reference overcome the temporal segment limitations of traditional window analysis, ensuring dynamic comparability of cross-period data spanning 2000-2022, thus avoiding inconsistencies in efficiency evaluation standards across different time periods. Finally, environmentally-adjusted super-efficiency scores enable precise identification of technically inefficient decision-making units,

providing a targeted basis for differentiated policy formulation. This integration significantly transcends the research paradigms in existing literature that employ single models or partial improvements, achieving a triple advancement: from single environmental factors to full life cycle in assessment dimensions; from static cross-sections to dynamic evolution in temporal scope; and from general recommendations to differentiated guidance in policy precision.
2. The findings discuss positive spatial spillovers, but the underlying causal mechanisms remain underdeveloped. Clarifying how policies or technologies diffuse across provinces would strengthen policy relevance.
**【Revision Notes】** Thank you very much for your valuable comments on this paper. We have added a detailed explanation of the three major transmission paths in the discussion section on spatial spillover effects. The modifications are primarily concentrated in section 4.2.2, the spatial spillover effect analysis section, which clearly articulates the three major transmission paths of spatial spillover effects: the technology diffusion effect, the policy imitation effect, and the factor mobility effect. We have also illustrated the actual operation of each transmission path through specific examples. Simultaneously, we have supplemented the "distance decay" characteristic of spatial spillover, providing a spatial scale decision-making reference for building regional collaborative mechanisms and enhancing the policy relevance of the research findings.<br>To make the overall structure of the paper more coherent, we have also added corresponding content to the conclusion section, summarizing the methodological integration and spatial spillover effect as our two key innovative points. The revisions are focused in section six (Conclusion and Recommendations), emphasizing that the innovative integration of LCA and the global super-efficiency SBM model expands the research perspective on the ecological efficiency evaluation of food production, and that the spatial spillover effects and their transmission mechanisms revealed in the study are of great significance for formulating regional collaborative policies.<br>Through the above revisions, we have emphasized the value of the methodological integration of this study, improved the causal mechanism explanation of spatial spillover effects, and enhanced the theoretical innovation and policy practical significance of the research results. The modifications not only address your key suggestions but also further improve the academic quality and application value of the paper.<br>**【Specific modifications are as follows】**<br>Spatial spillover effects primarily originate through three major transmission pathways: First, technology

diffusion effects, whereby conservation tillage techniques and precision fertilization techniques developed in major grain-producing regions spread to surrounding areas through agricultural technology extension systems. For example, black soil conservation techniques from Heilongjiang's agricultural reclamation system have been successfully promoted to neighboring provinces such as Jilin and Liaoning, generating significant technological spillovers. Second, policy emulation effects, primarily manifested as successful policy practices from high-efficiency regions being adapted and adopted by surrounding areas. For instance, Jiangsu Province's high-standard farmland construction practices have been rapidly emulated by adjacent provinces like Anhui and Zhejiang, forming regional policy learning networks. Third, factor mobility effects, whereby the movement of new-type professional farmers and entrepreneurs across regions facilitates the transmission of management expertise and production models, promoting the diffusion of environmentally friendly agricultural practices. Empirical studies indicate that these spillovers exhibit a clear “distance-decay” pattern, with high-efficiency regions typically forming “growth poles” for efficiency enhancement, whose influence diminishes as distance increases. This finding offers spatial-scale insights for constructing regional collaborative mechanisms, and accounts for why certain adjacent provinces exhibit patterns of “club convergence” in ecological efficiency.

3. Some sections methodology are dense with formulas and technical terms. The authors could add interpretative explanations to help general readers grasp the model outputs and implications more easily.

【Revision Notes】 Thank you very much for your valuable feedback on this paper. We have made the following revisions: First, in Section 2.2, we have added a more accessible explanation of the core formula of the Global Super-Efficiency SBM model, establishing a clear connection between mathematical symbols and their practical meanings. For example, for the efficiency value calculation formula, we added an explanation that it represents "the optimal input factor ratio for a decision-making unit to maintain its current output level," making it easier for readers to understand the actual meaning of the efficiency value. Second, we added a section on interpreting the model results, providing a detailed explanation of the meaning of the efficiency value range (0~1 or greater than 1). We explained the specific economic meanings of "technically efficient" and "technically inefficient," as well as the difference between super-efficiency values and conventional efficiency values. Third, we have added annotations to professional terms such as Spatial Kernel Density Estimation, making it easier for readers to understand the analysis results of distributional dynamic evolution.

These revisions significantly improve the readability of the methodology section, enabling readers

without a professional background to understand the basic principles of the model and the practical implications of the research results, while maintaining academic professionalism and rigor. We believe that these improvements will facilitate a broader readership's understanding and application of the findings of this study.

【Specific modifications are as follows】

2.2. Global super-efficient SBM model

This study employs a global super-efficiency Slacks-Based Measure (SBM) model, incorporating undesirable outputs, to assess grain production eco-efficiency. The methodology, based on Tone (2001) [42], involves two steps: Calculating efficiency scores using the global standard efficiency SBM model with undesirable outputs for all decision-making units (DMUs). Applying the global super-efficiency SBM model with undesirable outputs for effective DMUs. Our research focuses on this global super-efficiency SBM model, which accounts for non-expected outputs in grain production.

Assuming that the efficiency values of n DMUs are to be evaluated, for any DMU_j (j=1,2,...,n) in period t (t=1,2,...,T), all of them are co-produced using m inputs $x=(x_1, x_2,...,x_m)^T \in R^{m+}$, and co-produced with q outputs $y=(y_1, y_2,...,yq)^T \in R_q^+$ and h kinds of undesired outputs $b=(b_1, b_2,...,b_h)^T \in R_h^+$. Drawing on the work of Huang et al. (2014) [43], the following mathematical formulation quantifies the eco-efficiency of cereal cultivation.

$$
\begin{aligned}
&\rho^* = \min_{\lambda,s^-,s^+} \frac{1+\frac{1}{m}\sum_{i=1}^{m}\frac{s_{io}^{-,\tau}}{x_{io}^{\tau}}}{1-\frac{1}{q+h}\left(\sum_{r=1}^{q}\frac{s_{ro}^{+,\tau}}{y_{ro}^{\tau}}+\sum_{k=1}^{h}\frac{s_{ko}^{-,\tau}}{b_{ko}^{\tau}}\right)} \\
&s.t.\ \ x_{io}^{\tau} \ge \sum_{t=1}^{T}\sum_{j=1(j\ne o \text{ if } t=\tau)}^{n}\lambda_j^t x_{ij}^t - s_{io}^{-,\tau};\ i=1,2,\ldots,m; \\
&y_{ro}^{\tau} \le \sum_{t=1}^{T}\sum_{j=1(j\ne o \text{ if } t=\tau)}^{n}\lambda_j^t y_{rj}^t + s_{ro}^{+,\tau};\ r=1,2,\ldots,q; \\
&b_{ko}^{\tau} \ge \sum_{t=1}^{T}\sum_{j=1(j\ne o \text{ if } t=\tau)}^{n}\lambda_j^t b_{kj}^t - s_{ko}^{-,\tau};\ k=1,2,\ldots,h; \\
&1-\frac{1}{q+h}\left(\sum_{r=1}^{q}\frac{s_{ro}^{+,\tau}}{y_{ro}^{\tau}}+\sum_{k=1}^{h}\frac{s_{ko}^{-,\tau}}{b_{ko}^{\tau}}\right) \ge \varepsilon;\ \lambda_j^t \ge 0; \\
&s_{io}^{-,\tau},\ s_{ro}^{+,\tau},\ s_{ko}^{-,\tau} \ge 0;\ t=1,2,\ldots,T;\ j=1,2,\ldots,n(j\ne o \text{ if } t=\tau)
\end{aligned}
\tag{2}
$$

In the formula above, ρ* represents the efficiency score of the decision-making unit (DMU), with its economic interpretation being the proportion of input resources that can be reduced while maintaining the current output level. Specifically, the efficiency score ρ* derived from this model has a range of ρ* ≥ 1. If ρ* = 1, it indicates that the DMU is positioned on the optimal production frontier and is technically

efficient. If $\rho^* < 1$, it suggests that technical inefficiency exists within the DMU; the lower the efficiency score, the higher the degree of technical inefficiency and the greater the potential for improvement. If $\rho^* > 1$ (the super-efficiency case), it demonstrates that the DMU is located beyond the production frontier, with its technical efficiency exceeding the average level among the sample.

The efficiency scores derived from this model carry significant practical implications. Taking the efficiency score of 0.682 as an example, this indicates that China's grain production systems could, on average, reduce input resources (such as fertilizers, pesticides, water resources, etc.) by 31.8% while maintaining current grain output levels. The economic interpretations across different efficiency intervals are as follows: For scores < 0.5: This signifies severe resource waste and negative environmental externalities in the region's grain production system, with potential for at least a 50% reduction in resource inputs and pollution emissions; For 0.5 ⩽ scores < 0.8: This represents moderate efficiency levels, with substantial room for improvement in resource utilization and environmental protection; For 0.8 ⩽ scores < 1: This indicates relatively high efficiency levels, though minor improvements are still possible; For scores = 1: This demonstrates complete technical efficiency, with resource utilization reaching optimal levels; For scores > 1: This reflects a super-efficiency case, where the region's production technology and management practices outperform those of other regions.

This research achieves a triple methodological breakthrough by integrating Life Cycle Assessment (LCA) with a Global Super-efficiency SBM model: Firstly, the introduction of LCA addresses the deficiency of traditional DEA models in quantifying undesirable outputs such as non-point source pollution and carbon emissions, establishing a “sowing-to-harvest” full-chain environmental footprint assessment system that enables comprehensive quantification of ecological impacts throughout the entire grain production process, rather than merely focusing on localized environmental effects of specific stages. Secondly, efficiency measurements with global reference overcome the temporal segment limitations of traditional window analysis, ensuring dynamic comparability of cross-period data spanning 2000-2022, thus avoiding inconsistencies in efficiency evaluation standards across different time periods. Finally, environmentally-adjusted super-efficiency scores enable precise identification of technically inefficient decision-making units, providing a targeted basis for differentiated policy formulation. This integration significantly transcends the research paradigms in existing literature that employ single models or partial improvements, achieving a triple advancement: from single environmental factors to full life cycle in assessment dimensions; from static cross-

sections to dynamic evolution in temporal scope; and from general recommendations to differentiated guidance in policy precision.

2.3. Methods for analyzing the evolution of spatio-temporal dynamics

Kernel density estimation (KDE) is a non-parametric technique visualizing the dynamic evolution of grain production eco-efficiency distribution. The traditional KDE method primarily analyzes spatial distribution of non-equilibrium phenomena. The spatial KDE incorporates geographic factors, revealing long-term regional transfer trends. This study applies spatial KDE to agricultural eco-efficiency analysis. Using grain production eco-efficiency values, we categorize each region and its neighbors into four distinct groups, providing insights into spatial eco-efficiency patterns. Regions exhibiting a ratio below 25% of the highest-level region in the country are classified as exhibiting low efficiency. Regions exhibiting a ratio between 25% and 50% are classified as exhibiting medium-low efficiency, while those exhibiting a ratio between 50% and 75% are classified as exhibiting medium-high efficiency. Regions with a ratio exceeding 75% are considered to exhibit high efficiency. Equation (3) defines the conventional kernel density estimation, while its spatial counterpart is formulated in equation (4).

$$f(x)=\frac{1}{nh}\sum_{i=1}^{n}K(\frac{x_i-x}{h})$$
$$K(\mathrm{x})=\frac{1}{\sqrt{2\pi}}\exp(-\frac{x^2}{2}) \quad (3)$$

$$\mathrm{f}(x,y)=\frac{1}{nh_xh_y}\sum_{i=1}^{n}K_x\left(\frac{X_i-x}{h_x}\right)K_y\left(\frac{Y_i-y}{h_y}\right) \quad (4)$$
$$g(y|x)=\frac{f(x,y)}{f(x)}$$

In equation (3), f(x) denotes the probability density function, where xi represents the provincial cereal production eco-efficiency, and xi signifies its national mean. The variable n indicates the number of regions studied, h is the bandwidth parameter, and K symbolizes the Gaussian kernel function. Equation (4) introduces f(x,y) as the bivariate probability density, with g(y|x) representing the spatially conditioned kernel density.

4. The manuscript lacks a thorough discussion of data limitations, particularly the assumptions made in emission coefficients and the accuracy of provincial data over 23 years.

【Revision Notes】 Thank you for your valuable comments on this manuscript. We have made the following revisions:

1. In Section 2.5.3, we added a new subsection titled "Discussion of Data Limitations" to analyze potential limitations of the data used in this study. We explicitly state the sources of the emission factors applied for calculating carbon emissions and nonpoint source pollution, and we point out that regional differences in climate conditions, soil characteristics, and agricultural practices may cause spatial variability in those emission factors. We provide a detailed analysis of how these assumptions might potentially affect the study' s results, and we discuss possible changes in statistical definitions and measures that could affect the long time series data from 2000 - 2022.

2. In the Conclusion, we added a paragraph reflecting on the study's limitations, explicitly noting how data limitations may influence the generalizability and applicability of the findings, and offering recommendations for future research to further improve data quality.

These revisions improve the readability of the methodology section, making it easier for general readers to understand the model and the meaning of its outputs; at the same time, they add a systematic discussion of data limitations, enabling a more objective and accurate interpretation of the results. These changes help enhance the paper's scholarly rigor and practical value.

【Specific modifications are as follows】

2.5.3 Discussion of Data Limitations

While the data used in this study were derived from reliable sources, certain limitations exist, primarily manifested in two aspects: First, the issue of regional heterogeneity in emission factors. Although we explicitly utilized authoritative emission factors provided by IPCC, Tian and Zhang (2013), and Li et al. (2016) [40,41], the application of these coefficients across national scales may overlook natural environmental variations between regions. For instance, differences in soil physicochemical properties between northern and southern regions (such as organic matter content, pH value, etc.) may lead to variations in emissions under identical inputs; temperature and humidity differences across climate zones (temperate, subtropical, tropical) affect the formation mechanisms of CH_4 and N_2O, thereby influencing the accuracy of emission factors. During the study period, agricultural technological advancements may have altered certain emission factors. For example, the promotion of novel slow-release fertilizers may have reduced carbon emission intensity per unit of chemical fertilizer; updates in rice varieties and improvements in irrigation technologies may have affected CH_4 emission factors. The fixed emission factors employed in this study may underestimate the contribution of technological progress to emission reduction. Second, the accuracy issue with long-term

provincial data series. This research utilized 23-year provincial panel data spanning 2000-2022. Throughout this period, China's statistical system underwent multiple adjustments, with changes in statistical standards and calculation methodologies for certain indicators, potentially affecting the longitudinal comparability of the data.

Despite the meaningful findings generated by this study, certain limitations remain. First, the assumption of nationally uniform emission coefficients may underestimate the effects of regional heterogeneity. Second, long time-series data may be subject to inconsistencies in statistical methodologies and measurement errors. Finally, provincial-level analysis may mask efficiency disparities among different areas within provinces. Future research could enhance study quality by developing regionally differentiated emission coefficient systems and employing finer-grained data at municipal and county levels.

5. The conclusions are broad (e.g., promoting green agriculture), but specific, actionable recommendations for different regional categories (e.g., grain-consuming vs. producing provinces) would greatly enhance the utility of the study.

【Revision Notes】 Thank you very much for your valuable feedback on this article. We have carefully studied your review comments and made targeted revisions and improvements to the conclusions and recommendations section of the paper. The main modifications are as follows:

First, we have completely restructured the recommendations section, adopting a "category-based guidance, region-specific policy" framework. We have proposed differentiated and specific policy recommendations for three types of functional areas: major production areas, major consumption areas, and production-consumption balanced areas, avoiding the original vague expression of promoting green agriculture in a generalized manner. Second, based on the original single recommendation, we have added two dimensions: "regional coordination, overall improvement" and "policy coordination, systematic advancement." We have proposed systematic recommendations from the aspects of coordinating mechanism construction, the establishment of technology promotion systems, and the improvement of policy support systems, strengthening coordinated regional development. Third, we present the expected effects of implementing the above recommendations, including reducing agricultural carbon emission intensity, improving fertilizer and pesticide utilization rates, and reducing agricultural non-point source pollutant emissions, providing clear evaluation goals for policy implementation.

Through the above modifications, the conclusions and recommendations section of this paper highlights

the policy and practical significance, and the recommendations are more specific and actionable. The differentiated development strategies for different regional categories are clearer, effectively addressing your valuable comments. The revised content more fully reflects the applied value and practical significance of this research, providing more targeted policy references for promoting the ecological efficiency of grain production in China.

【Specific modifications are as follows】

6.2 Recommendations

The findings of the research study lead to the following recommendations:

First, Categorized guidance and region-specific implementation. (a) Major Production Areas: Leveraging green scale effects and consolidating ecological efficiency advantages. Establish agricultural technology integration demonstration bases in Heilongjiang, Jilin, and other regions; promote precision fertilization techniques; implement large-scale black soil conservation tillage; and establish crop rotation and fallow systems, allocating appropriate quantities of cropland for fallowing. (b) Major Consumption Areas: Developing high-quality, high-efficiency agriculture and accelerating ecological transformation. Construct several urban modern agricultural demonstration zones in Guangdong, Zhejiang, and other regions to improve output efficiency per unit area. (c) Production-Consumption Balanced Areas: Developing specialty ecological agriculture and constructing circular agricultural systems. Develop multiple specialty agricultural demonstration parks in Guangxi, Yunnan, and other regions to increase the growth rate of output value from specialty agricultural products.

Second, Regional coordination for holistic improvement. Establish a regional coordination mechanism led by the Ministry of Agriculture and Rural Affairs; convene regular joint conferences on collaborative development among the three functional area types; establish dedicated funds for regional ecological efficiency collaboration; and incorporate grain production ecological efficiency into government performance evaluations. Form a national expert committee on enhancing grain production ecological efficiency; implement the "Science and Technology Commissioner Plus" initiative, deploying numerous technical personnel to serve on the front lines; establish an agricultural technology training system to conduct large-scale training programs for new-type professional farmers.

Third, policy coordination for systematic advancement. Establish a green and low-carbon agriculture subsidy system that incorporates reduction and efficiency enhancement into subsidy criteria; reform

agricultural insurance policies by developing green crop insurance products; and establish an agricultural carbon sink trading mechanism. Continuously expand the area through high-efficiency water-saving irrigation; construct multiple regional grain logistics centers; and develop numerous rural clean energy demonstration villages. Establish a national green agriculture development fund; develop green agricultural credit products; and encourage the issuance of specialized green agricultural bonds. Implementation of these recommendations would likely ensure national food security while simultaneously reducing agricultural carbon emission intensity, improving utilization rates of fertilizers and pesticides, decreasing agricultural non-point source pollutant emissions, and providing robust support for green and low-carbon agricultural development.
